# Fcγ Receptors Contribute to the Antiviral Properties of Influenza Virus Neuraminidase-Specific Antibodies

**DOI:** 10.1128/mBio.01667-19

**Published:** 2019-10-22

**Authors:** E. R. Job, T. Ysenbaert, A. Smet, A. Van Hecke, L. Meuris, H. Kleanthous, X. Saelens, T. U. Vogel

**Affiliations:** a VIB-UGent Center for Medical Biotechnology, VIB, Ghent, Belgium; b Department of Biomedical Molecular Biology, Ghent University, Ghent, Belgium; c Department of Biochemistry and Microbiology, Ghent University, Ghent, Belgium; d Sanofi Pasteur, Research North America, Cambridge, Massachusetts, USA; Icahn School of Medicine at Mount Sinai

**Keywords:** influenza, neuraminidase, Fcγ receptor, antibodies, influenza vaccines

## Abstract

Influenza virus neuraminidase (NA) has been under intense study recently as a vaccine antigen, yet there remain unanswered questions regarding the immune response directed toward NA. Antibodies (Abs) that can inhibit NA activity have been shown to aid in the control of disease caused by influenza virus infection in humans and animal models, yet how and if interactions between the Fc portion of anti-NA Abs and Fcγ receptors (FcγR) contribute to protection has not yet been extensively studied. Herein, we show that poly- and monoclonal anti-NA IgG antibodies with NA inhibitory activity can control A(H1N1)pdm09 infection in the absence of FcγRs, but FcγR interaction aided in viral clearance from the lungs. In contrast, a mouse-human chimeric anti-NA IgG1 that was incapable of mediating NA inhibition (NI) solely relied on FcγR interaction to protect transgenic mice (with a humanized FcγR compartment) against A(H1N1)pdm09 infection. As such, this study suggests that NA-specific antibodies contribute to protection against influenza A virus infection even in the absence of NI activity and supports protection through multiple effector mechanisms.

## Abstract (precis)

There is a pressing need for next-generation influenza vaccine strategies that are better able to manage antigenic drift and the cocirculation of multiple drift variants and that consistently improve vaccine effectiveness. Influenza virus NA is a key target antigen as a component of a next-generation vaccine in the influenza field, with evidence for a role in protective immunity in humans. However, mechanisms of protection provided by antibodies directed to NA remain largely unexplored. Herein, we show that antibody Fc interaction with Fcγ receptors (FcγRs) expressed on effector cells contributes to viral control in a murine model of influenza. Importantly, a chimeric mouse-human IgG1 with no direct antiviral activity was demonstrated to solely rely on FcγRs to protect mice from disease. Therefore, antibodies without NA enzymatic inhibitory activity may also play a role in controlling influenza viruses and should be of consideration when designing NA-based vaccines and assessing immunogenicity.

## INTRODUCTION

Despite years of intensive research, influenza remains a public health burden worldwide. The current split inactivated influenza vaccines (IIV) available are aimed at inducing neutralizing antibodies (Abs) mainly directed toward hemagglutinin (HA), the major glycoprotein on the surface of the virus. While vaccine effectiveness is 50 to 60% when the strains in the IIV match the antigenicity of the circulating strain (1), it can drop to as little as 10% when there is no match (or less than 10%, depending on season and country), as demonstrated in the 2004-2005 Northern Hemisphere influenza season (2). As such, the scientific community is investing large efforts to try to increase influenza vaccine efficacy by various approaches (reviewed in references 3 and 4).

Neuraminidase (NA) is the second major glycoprotein of influenza type A (IAV) and B viruses. NA exists as a tetramer on the surface of the virion and infected cells. Whereas HA binds to sialic acid on the surface of target cells, an essential step to initiate infection, NA has enzymatic activity to cleave sialic acid from the underlying galactose residues (5) and promote viral spread. In recent years, NA has regained attention as a vaccine candidate. However, unlike HA, much less is known about the antigenic relatedness or landscape of NAs and the protective mechanisms of anti-NA antibodies. Antibodies directed toward the active site of the NA are able to block catalytic activity (6) and studies have shown these antibodies are capable of ameliorating disease in mice (7), ferrets (8, 9), and humans (10). Further, studies have identified antibodies that can bind to NA at a site distant from the active site yet are still able to inhibit enzymatic activity and protect mice (11).

In addition to direct antiviral activities, the antibody Fc domain can interact with Fcγ receptors (FcγRs) expressed on a number of cells, including macrophages, dendritic cells (DCs), and natural killer (NK) cells. This interaction may contribute to viral clearance and promote uptake of immune complexes (reviewed in reference 12). Influenza virus HA stalk (13) and M2e-specific (14) antibodies, for the most part, rely on interactions with FcγR to mediate optimal effector function. For NA Abs, the interaction with FcγRs is largely underexplored. Historical studies by Tamura et al. describe the ability of polyclonal anti-NA sera to increase uptake of N1 and N2 viruses by the murine macrophage FcγR^+^ cell line P388D (15). DiLillo et al. (13) reported that the broadly binding human IgG1 anti-NA monoclonal antibody 3C05, with specificity shown for A(H1N1)pdm09 and the seasonal H1N1 virus A/Brisbane/07, relied on FcγR interaction for protection against influenza in mice, while an IgG1 antibody with specificity only for an A(H1N1)pdm09 strain, 3C02, did not. However, these antibodies have been poorly defined in literature, and little is known, to our knowledge, beyond their ability to bind and neutralize H1N1 influenza viruses (13). Antibodies directed toward the NA protein also mediate antibody-dependent cellular cytotoxicity (ADCC) *in vitro*, a mechanism reliant on FcγR engagement (16, 17). Furthermore, NA inhibition (NI) antibodies can cooperatively increase ADCC together with HA stalk antibodies but display weak induction of ADCC alone (18). Whether this contributes to protection *in vivo* is unknown. Kim et al. (19) have shown that FcγR knockout (KO) mice lose slightly more weight than wild-type (WT) counterparts when infected with A(H1N1)pdm09 preincubated with polyclonal sera raised to recombinant N1 NA. Finally, Kawaoka and colleagues recently demonstrated that human A(H1N1)pdm09 anti-NA antibodies that target the lateral side of the NA head not only protect in an Fc-dependent manner but may also drive antigenic drift in the lateral portion of the NA head (20).

Herein, we show that Fc-FcγR engagement is not required for protection provided by a polyclonal NI serum in mice. Further, we demonstrate that an A(H1N1)pdm09 NA-specific monoclonal antibody, N1-C4, that is capable of blocking viral NA activity can control A(H1N1)pdm09 infection in the absence of FcγRs, but FcγRs are important for the clearance of virus from the lungs. This is in contrast to a chimeric human IgG1 monoclonal antibody, huN1-7D3, that can bind to A(H1N1)pdm09 but does not possess direct antiviral activity, with control of influenza virus infection relying on engagement of FcγRs.

## RESULTS

### Protection of mice with antibodies that are capable of mediating NI does not solely rely on FcγR interaction to protect against influenza disease.

**FIG 1:**
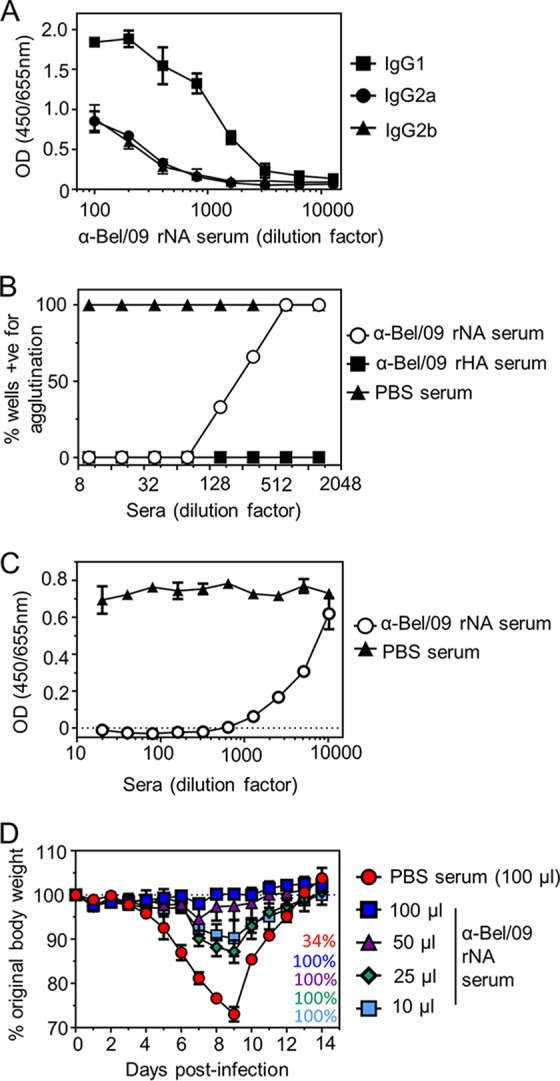
Characterization of polyclonal anti-rNA immune serum *in vitro* and *in vivo*. (A) Binding of mouse antisera to Bel/09 rNA. Wells of a 96-well flat-bottom ELISA plate were coated with 0.5 μg/ml of Bel/09 rNA overnight in sodium carbonate buffer. Wells were then blocked with 1% BSA in PBS, and heat-inactivated antiserum was subsequently applied in serial dilutions and incubated for 2 h at room temperature in 0.5 mg/ml BSA in PBST. Binding of antisera was detected with either anti-mouse IgG1, IgG2a, or IgG2b conjugated to HRP. Data represents the average from 2 independent experiments ± standard deviation (SD). OD, optical density. (B) Ability of antisera to inhibit virus infectivity. One hundred TCID_50_ of Bel/09 was preincubated with increasing dilutions of heat-inactivated antisera directed toward rNA or rHA of Bel/09 or preimmune serum in triplicate and subsequently added to prewashed confluent monolayers of MDCKs. Cells were incubated for 6 days in the presence of trypsin. Virus replication was detected by a hemagglutination assay with turkey red blood cells. Results are expressed as the percentage of wells that scored positive for agglutination. (C) Antisera directed toward Bel/09 rNA can inhibit the viral NA activity. Bel/09 was incubated with increasing dilutions of heat-inactivated anti-Bel/09 NA sera or PBS antisera, and NA activity was determined at 18 h postincubation on fetuin as described in Materials and Methods. Data represent at least 2 independent repeats and show the average ± SD from one experiment performed in duplicate. (D) Anti-Bel/09 NA serum protects mice in a dose-dependent manner. Mice were treated via the i.p. route with 100 μl of heat-inactivated mock serum or increasing amounts of anti-Bel/09 rNA serum and subsequently infected with 1 LD_50_ of Bel/09. Mice were monitored for 14 days for weight loss. Mice were sacrificed if they had lost ≥25% of their original body weight. Survival percentages are indicated on the bottom right-hand side of the graph. Text colors match the legend color for each group. Data are representative of 2 repeats and show the average weight loss over time ± the standard error of the mean (SEM [*n* = 3]).

Previous studies have shown that Fc-FcγR interactions contribute little to the protection of mice treated with anti-A(H1N1)pdm09 polyclonal serum against homologous challenge (19). Initially, we sought to confirm this phenotype in the context of a protective polyclonal anti-NA immune serum. Polyclonal serum was raised to adjuvanted soluble tetrameric NA (rNA), derived from H1N1 A/Belgium/1/2009, in BALB/c mice. The resulting immune serum was heat inactivated and characterized *in vitro* and *in vivo* (Fig. 1). Vaccination raised a mixture of IgG isotypes directed toward the rNA, including IgG1, IgG2a, and IgG2b, with IgG1 showing the strongest signal in an enzyme-linked immunosorbent assay (ELISA), whereas IgG3 and IgE were undetectable by ELISA (Fig. 1A and data not shown). Next, the ability of the serum to neutralize infectivity of the mouse-adapted descendant of A/Belgium/1/2009 (named Bel/09) was tested. Serum raised to buffer with adjuvant (phosphate-buffered saline [PBS]) was unable to neutralize virus infectivity, whereas anti-recombinant soluble Bel/09 HA (rHA) immune serum neutralized the virus at all concentrations tested. For antiserum directed against rNA, there was a titratable effect on viral infectivity (Fig. 1B), in line with previous studies (21). Further, the anti-Bel/09 NA serum was also able to inhibit the viral NA activity in an NA inhibition (NI) assay, whereas the PBS serum could not (Fig. 1C). Finally, to determine an *in vivo* protective dose, BALB/c mice were treated via the intraperitoneal (i.p.) route with either PBS serum as a negative control (100 μl) or increasing amounts of anti-Bel/09 rNA serum. One day later, the mice were infected with 1 50% lethal dose (LD_50_) of Bel/09 and monitored for weight loss over 14 days. All mice treated with anti-NA serum were significantly protected from weight loss in comparison to mock serum-treated mice (*P* < 0.05, two-way analysis of variance [ANOVA], main column effects). Fifty microliters of rNA immune serum was selected for further studies, as this conserved the antiserum and showed almost 100% protection from weight loss (Fig. 1D).

**FIG 2:**
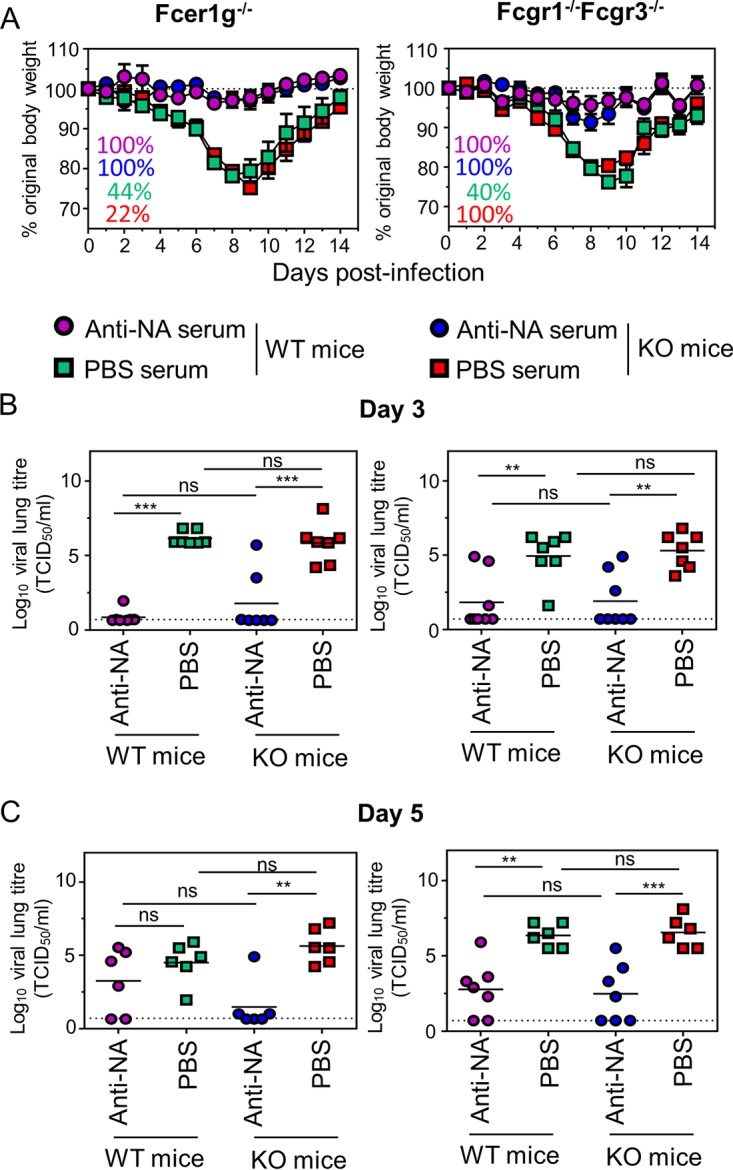
Activating FcγRs are not required to control influenza virus in mice treated with polyclonal anti-NA serum. Fifty microliters of heat-inactivated anti-NA sera (circles) or PBS antisera (squares) were passively transferred via the i.p. route into *Fcer1g* or *Fcgr1/Fcgr3* knockout (KO) mice. BALB/c WT mice were included as controls. One day later, the mice were challenged with 1 LD_50_ of Bel/09 and monitored daily, and any mice that had lost ≥25% of their original body weight were euthanized. Weight loss data represent the mean percentages ± SEM of original body weight over time (*n* = 8 to 10, pooled from 2 independent experiments). Survival percentages are indicated in the left-hand corner of the graphs where the text color matches colors indicated in the legend for each group (A). In a separate set of experiments, after passive transfer, KO or WT mice were challenged with 0.1 LD_50_ of Bel/09 and viral titers assessed in lung homogenates on day 3 (B) or day 5 (C) by TCID_50_. The horizontal lines represent the mean (pooled from 2 independent experiments). The dotted line indicates the detection limit of the TCID_50_. ns, nonsignificant. *, *P* < 0.05, **, *P* < 0.01, and ***, *P* < 0.001, by one-way ANOVA.

We next examined the possible contribution of FcγRs to the protection provided by anti-NA serum. Wild-type (WT), *Fcer1g*^−/−^ or *Fcgr1*^−/−^ *Fcgr3^−/−^* mice were pretreated i.p. with 50 μl of heat-inactivated anti-NA or PBS antiserum, as a negative control. *Fcer1g^−/−^* mice lack the common γ chain that, in mice, is required for the functional expression of FcγRI, -III, and –IV, whereas *Fcgr1^−/−^ Fcgr_3_^−/−^* mice are unable to express both FcγRI and -III. One day following immune serum administration, the mice were challenged with 1 LD_50_ of Bel/09 and monitored for weight loss over 14 days. WT, *Fcer1g^−/−^*, and *Fcgr1^−/−^ Fcgr_3_^−/−^* knockout (KO) mice treated with anti-NA sera were protected from weight loss and displayed no significant difference in weight loss over-time. WT and knockout mice treated with serum from the PBS-administered group displayed transient weight loss, with some mice succumbing to the infection (Fig. 2A). Importantly there was also no significant difference between challenged mock-treated WT and KO mice, suggesting that there was no intrinsic susceptibility difference between the WT and KO mice for influenza virulence (Fig. 2A). To examine the possible impact of FcγRs on the ability to control virus replication, lung viral loads were examined. Mice were treated as in the weight loss experiments and challenged 1 day later with 0.1 LD_50_ of Bel/09. On days 3 and 5 after infection, lung homogenates were assessed for the presence of infectious virus. Treatment of challenged mice with polyclonal anti-NA serum reduced viral titers in comparison to mock-treated mice in both WT and KO animals on days 3 (Fig. 2B) and 5 (Fig. 2C) postinfection. However, by day 5, at the dose tested in this passive serum transfer experiment, the strong reduction in viral titers observed in anti-NA-treated mice on day 3 was less pronounced. There was no significant difference between WT and KO mice in the control of virus replication on days 3 and 5. Taken together, these data show that polyclonal anti-NA responses with NI activity do not rely on FcγRs for protection against a homologous virus challenge in the mouse model.

### FcγRs contribute to the control of viral replication by a monoclonal antibody with NI activity.

**FIG 3:**
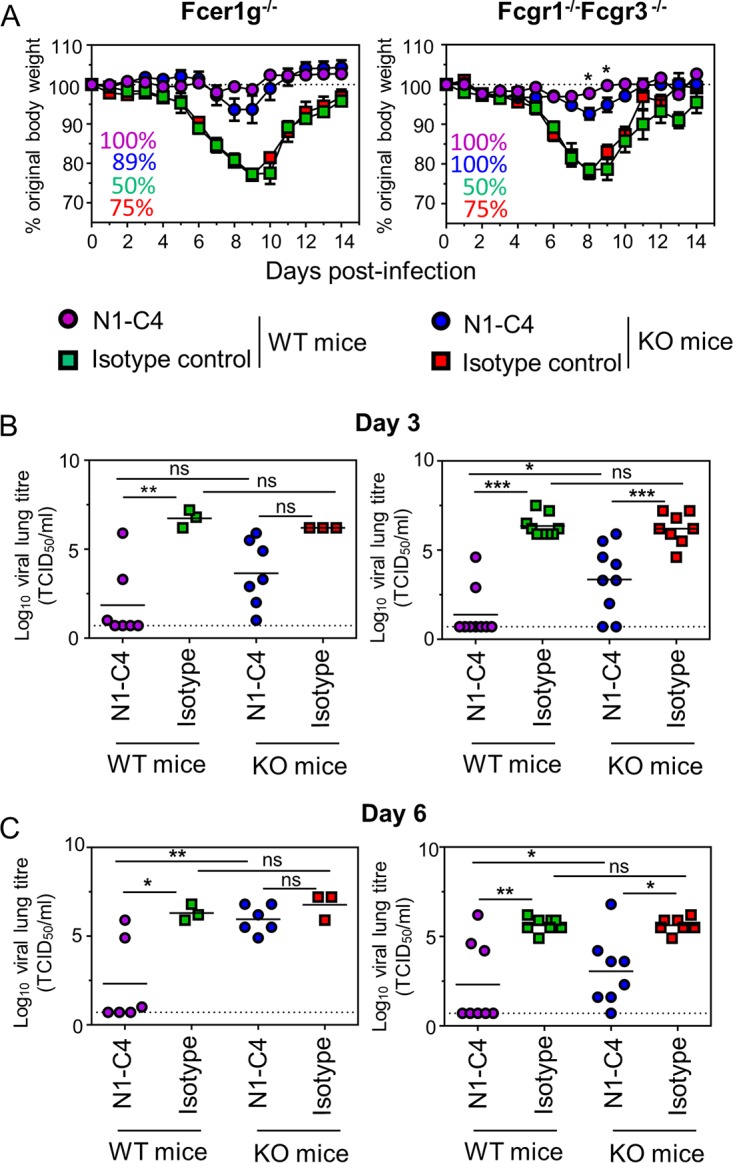
FcγRs aid in viral clearance but do not significantly impact weight loss in mice treated with N1-C4. *Fcer1g* or *Fcgr1/Fcgr3* KO mice (white symbols) were treated with 1 mg/kg of N1-C4 (circles) or a mouse IgG1 isotype control (squares) via the intranasal route under isoflurane sedation. BALB/c WT mice were included as controls (black symbols). One day later, the mice were challenged with 1 LD_50_ of Bel/09 and monitored daily, and any mice that had lost ≥25% of their original body weight were euthanized. Survival percentages are indicated on the left-hand side of the graphs, where the text color matches colors indicated for each group in the legend. Weight loss data represent the mean percentages ± SEM of original body weight over time (*n* = 8 to 10 pooled from 2 independent experiments) (A). In a separate set of experiments, after passive transfer, KO or WT mice were challenged with 0.1 LD_50_ of Bel/09, and viral titers were assessed in lung homogenates on day 3 (B) or day 6 (C) by TCID_50_. The horizontal lines represent the mean. The dotted line indicates the detection limit of the TCID_50_. ns, nonsignificant. *, *P* < 0.05, **, *P* < 0.01, and ***, *P* < 0.001, by one-way ANOVA.

Thus far, we have shown that a polyclonal anti-NA response does not rely on the engagement of FcγRs to control viral infection. As polyclonal responses are complex involving a variety of antibodies with different affinities for NA and comprises antibodies with and without NI activity, it was important to address if a monoclonal antibody directed against NA would result in the same outcome. Previously, we isolated and defined an A(H1N1)pdm09 N1-specific IgG1 mouse monoclonal antibody, N1-C4, with NI activity, which could control infection of Bel/09 virus in mice (7). We sought to address if N1-C4 could protect against influenza virus infection in the absence of activating FcγRs. WT, *Fcer1g*^−/−^, or *Fcgr1^−/−^ Fcgr3^−/−^* BALB/c mice were treated intranasally with 1 mg/kg of body weight N1-C4 or an isotype control. Twenty-four hours later, the animals were challenged with 1 LD_50_ of Bel/09 and monitored for weight change (Fig. 3A). Challenged isotype control-treated mice experienced transient severe weight loss with approximately half of the mice recovering from infection. There were no significant differences between KO and WT mice treated with the isotype control. N1-C4-treated WT, *Fcer1g^−/−^*, and *Fcgr1^−/−^ Fcgr3^−/−^* mice displayed minor weight loss with all mice recovering from infection. Interestingly, on days 8 and 9 knockout mice displayed a slightly higher weight loss than WT counterparts, although this only reached a statistically significant level in the *Fcgr1^−/−^ Fcgr_3_^−/−^* mice. Next, viral loads were determined in the lungs of mice treated with N1-C4 or the isotype control followed by infection with Bel/09 at 0.1 LD_50_. On day 3 postinfection in the N1-C4-treated *Fcer1g^−/−^* mice, there was a clear trend for increased levels of virus compared to WT mice (Fig. 3B, left-hand panel). This was also evident in the double-KO mouse strain, where the absence of FcRγI and FcγRIII significantly impacted the ability of the mice to control viral loads (*P* < 0.05 compared to WT mice, one-way ANOVA) (Fig. 3B, right-hand panel). This trend remained on day 6 postinfection with higher levels of virus in the KO compared to the WT N1-C4-treated mice (Fig. 3C). Thus, we conclude that N1-C4 is capable of controlling infection in mice in the absence of FcγRs; however, in the absence of the activating FcγRI and -III or in mice that lack the common γ chain, viral clearance within the lungs is less efficient than in WT mice.

### Development and characterization of a human IgG1 chimeric anti-NA antibody.

The data presented above have explored antibody responses with direct antiviral activity, and as such, it is not unreasonable that the antibodies do not rely solely on FcγRs to control an influenza virus infection. Recent studies have indeed shown that antibodies without NI activity can protect mice in an Fc-dependent manner (20). We have previously isolated a mouse IgG1 monoclonal antibody, N1-7D3, that could bind to Bel/09 NA but was unable to inhibit the viral NA activity or protect mice *in vivo*. As mouse IgG1 is poor at engaging activating FcγRs, interacting only with mouse FcγRIII (12), we next sought to enhance the affinity of N1-7D3 for FcγRs before assessing its protective ability. A chimeric antibody was constructed with the variable domains of mouse N1-7D3 and the constant regions of human IgG1 (here called huN1-7D3). Human IgG1 can interact with all human activating FcγRs. Likewise, N1-C4 was engineered to express human IgG1 constant domains (here named huN1-C4) and thus represents a chimeric human N1 NA-specific antibody that has direct antiviral activity.

**FIG 4:**
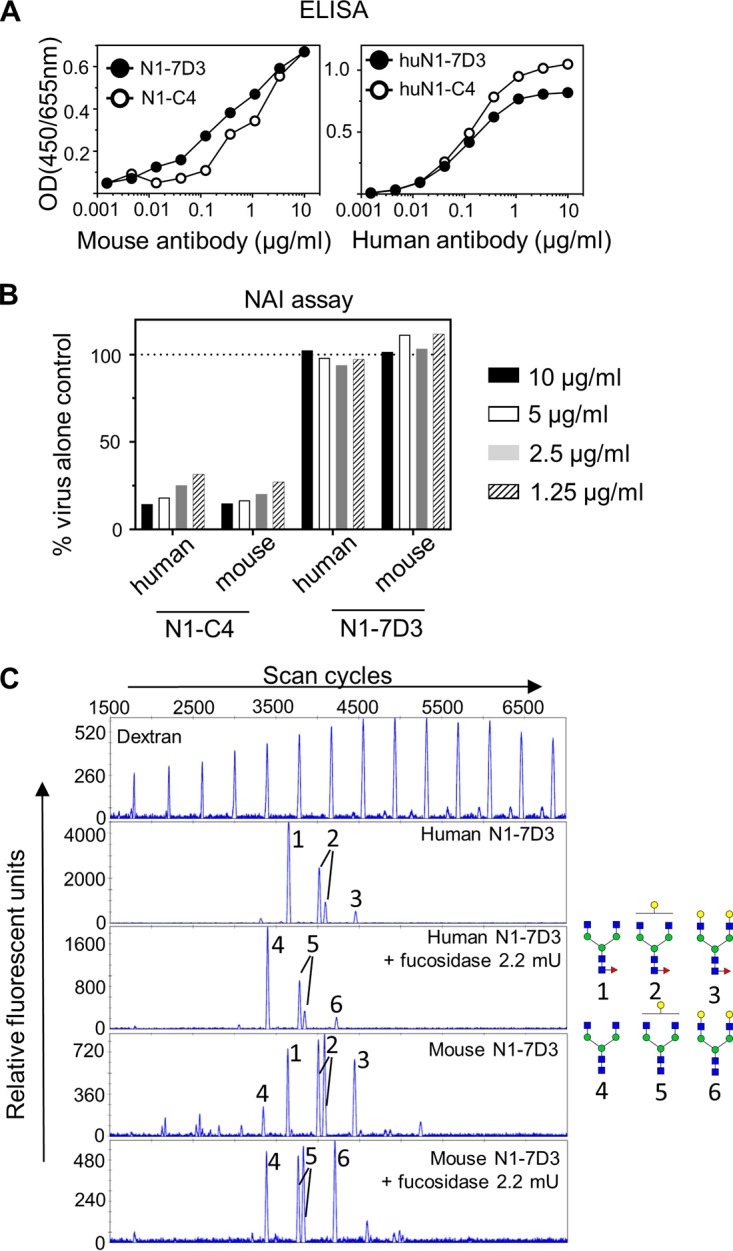
(A) HuN1-7D3 and huN1-C4 bind to recombinant NA from Bel/09. Wells of a 96-well ELISA plate were coated overnight with 0.5 μg/ml of recombinant soluble NA from Bel/09 in DPBS plus Ca^2+^/Mg^2+^. Plates were blocked with 1% BSA in PBS, and the mouse (left) or human chimera version of N1-7D3 or N1-C4 (right) was applied in increasing concentrations in 0.5% BSA in PBST. Binding was detected with either anti-mouse IgG or anti-human IgG conjugated to HRP as appropriate. OD, optical density. (B) HuN1-7D3 does not mediate NA activity against Bel/09, whereas huN1-C4 does. A constant amount of Bel/09 was incubated with 10, 5, 2.5, or 1.25 μg/ml of either huN1-C4, mouse N1-C4, huN1-7D3, or mouse N1-7D3, and NA activity was determined at 18 h postincubation on fetuin by an ELLA. Data are expressed as the percentage of the virus-alone control. NAI, NA inhibitor. (C) N-glycan profiling of mouse and humanized N1-7D3. N-glycans were isolated from mouse or humanized N1-7D3, labeled with APTS, either untreated or treated overnight with fucosidase, and analyzed by DNA sequencer-assisted fluorophore-assisted capillary electrophoresis. A labeled dextran ladder (top panel) and N-glycans from bovine RNase B (data not shown) were included as electrophoretic mobility references. The structures of the N-glycans that correspond to the indicated peaks are shown on the right. Blue squares, *N-*acetylglucosamine residues; green circles, mannose residues; yellow circles, galactose residues; red triangles, fucose residues.

As altering the constant region may impact the ability to bind to the antigen compared to the native antibody (22), we initially ascertained that huN1-7D3 and huN1-C4 behaved like the native mouse monoclonal antibodies. Similar to mouse N1-7D3 and N1-C4, huN1-7D3 and huN1-C4 were both able to bind to Bel/09 rNA in a dose-dependent manner (Fig. 4A). Furthermore, while human and mouse N1-C4 could block the NA activity of Bel/09, N1-7D3 (human and mouse) could not, consistent with our previous studies (Fig. 4B). Therefore, both huN1-C4 and huN1-7D3 behaved as expected.

It is known that core fucosylation of the conserved N-glycan at site Asn_297_ in the Fc tail of human IgGs modulates interaction with the FcγRIIIa on NK cells and the loss of fucose at this site results in a substantial increase in ADCC activity (23). We next used glycan profiling to compare the extent of core fucosylation of the chimeric and the native mouse monoclonal antibodies. N-glycans from the monoclonal antibodies were isolated by PNGase F treatment, labeled with 8-amino-1,3,6-pyrenetrisulfonic acid (APTS), and subsequently treated or not with fucosidase. The labeled glycans were then separated by capillary electrophoresis. A labeled dextran ladder and N-glycans from bovine RNase B were taken along as standards. Four major glycan peaks were identified in mouse and the human chimeric antibodies. Treatment of both N1-7D3 and huN1-7D3 with fucosidase resulted in a mobility shift of the 4 main peaks, indicating that the antibody glycans were almost fully core fucosylated (Fig. 4C), consistent with typical glycosylation forms found on Asn_297_ in wild type antibodies. Mouse and human chimeric N1-C4 were also both core fucosylated (data not shown). As such, it is expected that fucosylation at Asn_297_ will not contribute to any additional biological activity of huN1-7D3 compared to N1-7D3 produced by the mouse hybridoma.

### FcγR engagement is important for control of IAV infection by an NA-specific monoclonal antibody that lacks NI activity.

To examine if huN1-7D3 could possibly protect against an influenza virus infection *in vivo* we utilized the transgenic humanized FcγR mice (huFcγR mice), which are knocked out for mouse FcγRs and transgenic for all human Fcγ-activating receptors (24). Mice were treated intranasally (i.n.) with 2.5 mg/kg of huN1-C4, huN1-7D3, or a human IgG1 isotype control. One day later, the mice were challenged with Bel/09 at 1 LD_50_ and monitored for weight loss and survival. Both huN1-C4- and huN1-7D3-treated mice lost significantly less weight than the isotype-treated control mice, with 100% of the mice surviving the infection when treated with anti-NA monoclonal antibodies versus a 50% survival rate for the isotype control-treated mice. Hence, with an Fc that can engage multiple Fcγ-activating receptors, N1-7D3 now displayed the potential to protect against influenza virus infection in mice.

**FIG 5:**
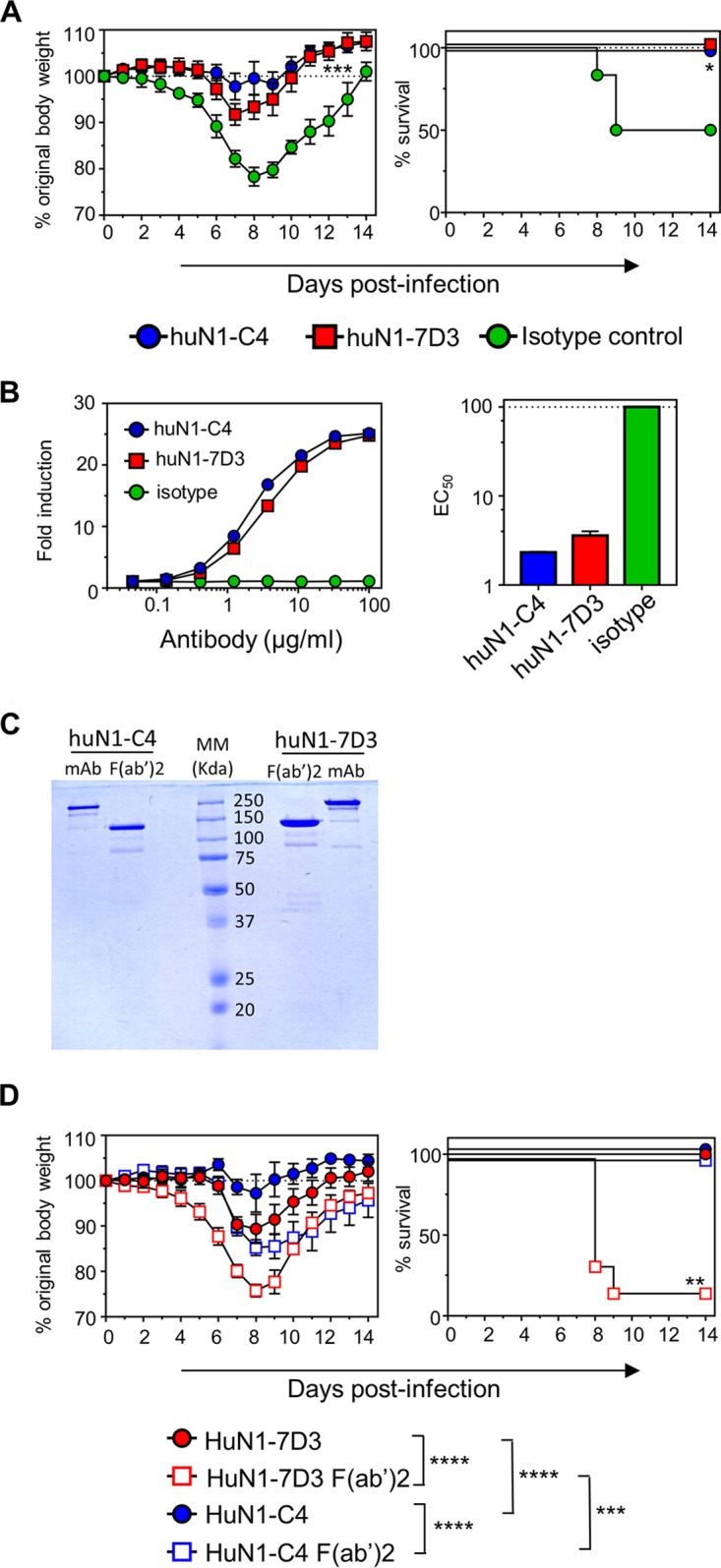
(A) HuN1-7D3 and huN1-C4 can protect against morbidity and mortality in humanized FcγR mice. Humanized FcγR mice were treated with 2.5 mg/kg μg of huN1-7D3, huN1-C4, or an isotype control via the i.n. route under isoflurane sedation. One day later, the mice were infected with 1 LD_50_ of Bel/09 and monitored daily over 14 days for weight loss (left panels) and survival (right panels). Weight loss data represents the mean percentage ± SEM of original body weight over time, and survival data are shown as the percentage of survival over time (*n* = 6 to 8). Weight loss over time was analyzed by two-way ANOVA examining the main column effects, and survival proportions were assessed using a two-tailed, log-rank (Mantel-Cox) test. *, *P* < 0.05, and ***, *P* < 0.001, in comparison to isotype-treated mice. (B) Human chimeric monoclonal antibodies can engage with FcγRIIIa expressed on a Jurkat reporter cell line. Bel/09-infected MDCK cells were used as target cells for a reporter assay where huN1-C4, huN1-7D3, and an isotype control were investigated for their ability to engage the FcγRIIIa expressed on the Jurkat cell line. Antibodies were tested in triplicate in 3-fold dilutions starting from a concentration of 100 μg/ml. Fold induction was calculated from the buffer-alone control (left panel), and curves were used to define EC_50_ values in GraphPad Prism (right panel). (C) Coomassie-stained nonreducing SDS-PAGE confirming the correct molecular weight of F(ab′)_2_ fragments. HuN1-C4 and huN1-7D3 were treated with IdeS enzyme using the FragIT kit according to the manufacturer’s instructions. Human monoclonal antibodies and F(ab′)_2_ fragments were run on a 10% SDS-PAGE alongside a molecular marker (MM) to resolve proteins according to their size. The F(ab′)_2_ fragment preparations do not contain intact uncleaved antibody. (D) HuN1-7D3 F(ab′)_2_ does not protect against Bel/09 infection in humanized FcγR mice. Humanized mice were treated with equimolar amounts of monoclonal antibodies (2.5 mg/kg) or F(ab′)_2_ fragments (1.85 mg/kg) in a total volume of 50 μl via the intranasal route 1 day prior to infection with 2 LD_50_s of Bel/09. Mice were monitored for weight loss (left panel) and survival (right panel) for 14 days. Mice that had lost ≥25% of their original body weight were euthanized. Weight loss data show the mean percentage ± SEM of original body weight over time and analyzed by a two-way ANOVA examining main column effects. ***, *P* < 0.001; ****, *P* < 0.0001. Survival data are the percentage of survival over 14 days (*n* = 6) and were assessed by a two-tailed, log-rank (Mantel-Cox) test. **, *P* < 0.01 compared to all other groups. Data were pooled from two individual experiments.

To explore a potential Fc-mediated mechanism of action for the protection provided by the human chimeric antibodies, an ADCC reporter assay was used. MDCK cells were infected with Bel/09 at a multiplicity of infection (MOI) of 1 and used as target cells for the antibodies to target NA expressed at the surface of the cells. Both huN1-C4 and huN1-7D3 were able to engage the FcγRIIIa eceptor expressed on Jurkat cells, resulting in a luciferase signal, whereas the isotype control could not (Fig. 5B).

Next, for more definitive evidence that huN1-7D3 controlled infection through engagement of FcγRs, the Fc portion of huN1-7D3 and huN1-C4 was removed by treatment with IdeS enzyme to create F(ab′)_2_ fragments (Fig. 5C). F(ab′)_2_ fragments and intact antibodies were subsequently passively transferred in huFγR mice at equimolar amounts (Fig. 5D). One day later, the mice were challenged with Bel/09 at 2 LD_50_s and monitored for weight loss and survival. As expected, all the mice treated with antibodies huN1-C4 and huN1-7D3 survived the challenge infection. However, huN1-C4-treated mice displayed significantly less weight loss than huN1-7D3 treated mice (*P* < 0.0001, two-way ANOVA, main column effects). The F(ab′)_2_ fragment of huN1-C4 also protected from lethality, albeit mice lost significantly more weight than mice treated with intact huN1-C4 monoclonal antibody (*P* < 0.0001, two-way ANOVA, main column effects). In contrast, the huN1-7D3 F(ab′)_2_ fragment was unable to protect the mice, where the majority of the mice succumbed to the infection by days 8 and 9. Therefore, we conclude that huN1-7D3 relies on Fc-FcγR interactions to provide protection in humanized mice.

## DISCUSSION

Fcγ effector functions have been shown to contribute to various aspects of antibody-mediated protection against influenza viruses, particularly for broadly binding antibodies. For example, the engagement of FcγR on alveolar macrophages has been shown to be crucial for M2e antibody-mediated protection (14). Furthermore, the majority of HA stem-specific antibodies require interaction with effector cells, including macrophages (18) and NK cells to control influenza virus infection *in vivo*. For NA antibodies, this area is ill-defined, with limited studies examining the contribution of Fcγ effector functions.

Kim et al. (19) reported that in the absence of FcγRs, anti-NA serum was still able to control infection with H1N1pdm09. Our results are in line with these studies where we observed no major difference in weight loss and survival over time in the presence of NI antiserum or monoclonal antibody N1-C4. Studies presented herein expanded on previous work by Kim et al. by examining the viral loads in the lungs at various days after infection. For polyclonal antiserum viral lung loads in FcγR-deficient mice were not significantly different from those in WT mice. However, FcγRs enhanced the ability of monoclonal antibody N1-C4 to control virus loads in the lungs, particularly on day 6 postinfection. Additionally, treating huFcγR mice with the F(ab′)_2_ fragment of huN1-C4 could still protect, but mice displayed significantly more weight loss than those treated with intact monoclonal huN1-C4, although in this case, we cannot rule out that a decreased half-life of the F(ab′)_2_ fragment, compared to the intact monoclonal antibody, contributed to the reduced protection. Besides ADCC and antibody-dependent cellular phagocytosis (ADCP) to aid in viral clearance, FcγRs have been implicated in maturation and activation of antigen-presenting cells (APCs) and in turn activation of T cells, plus in B cell maturation and affinity selection (reviewed by Pincetic et al. [25]). We speculate that while NI monoclonal antibodies aid in the initial control of the virus, engagement of FcγRs could play a role in enhancing viral clearance from the lungs.

We observed that *Fcer1g*^−/−^ mice treated with N1-C4 on day 6 postinfection displayed reduced viral clearance compared to *Fcgr1*^−/−^ *Fcgr3^−/−^* mice (Fig. 3C). *Fcer1g*^−/−^ mice lack the FcRγ chain, which associates not only with FcRs I, III, and IV but also with FcεRI and several other molecules. Of interest are members of the C-type lectin family—dendritic cell-associated C-type lectin-2 (Dectin-2), macrophage-inducible C-type lectin (Mincle), dendritic cell immunoactivating receptor (DCAR) and blood dendritic cell antigen 2 (BDCA-2)—all of which have been shown to function as pattern recognition receptors for various human pathogens (26). There is a clear role described for the interaction of influenza virus and other reported C-type lectins (27). In addition, Mincle has been shown to be upregulated during influenza virus infection (28).

By altering the Fc tail of N1-7D3 from a mouse IgG1 to one of human IgG, which can engage multiple human FcγRs, we were able to protect humanized FcγR mice against a potentially lethal challenge of H1N1pdm09. In line with this, by removing the Fc portion of huN1-7D3, protection was lost. As such it is clear that N1-7D3 relies on FcγR engagement to mediate protection, in contrast to N1-C4, which is able to control the virus by direct inhibition of viral NA activity. Glycan analysis revealed that both huN1-C4 and huN1-7D3 displayed core fucosylation at site Asn_297_. Engineering these monoclonal antibodies without a core fucose at this site would be of interest as studies have shown that this can significantly enhance ADCC activity (29), and thus, in turn could provide more potent protection against influenza virus. To our knowledge, core fucose mutants have never been investigated to increase protection against influenza virus; however, the GASD/ALIE Fc mutant of an anti-HA stalk antibody, 6F12, has been shown to provide significantly greater protection against PR8/34 infection in humanized FcγR mice compared to the wild-type antibody (30). This mutant shows enhanced binding to activating huFcγRs and increased activation of ADCC. The area of antibody engineering research holds great promise for the future development of potent anti-influenza antibodies.

DiLillo et al. (13) have previously shown that the broad anti-NA antibody 3C05 required the engagement of FcγRs to protect against H1N1pdm09, whereas a strain-specific antibody did not. N1-7D3 also broadly binds to N1 viruses at a conserved linear peptide at the carboxy terminus of the NA (7). Results presented here confirm the studies by DiLillo et al.; however, it remains to be seen if huN1-7D3 can also protect in mice against a variety of N1 IAVs. Furthermore, while we demonstrated that huN1-7D3 could engage with FcγRIIIa on a reporter cell line, the contribution of this engagement to mediate protection *in vivo* is unknown and whether other effector functions are involved is also unknown. Future studies are needed to address this. Importantly, these future studies also need to examine the possible synergy between HA and NA antibodies and whether increased induction of NA immunity by influenza virus vaccines can provide better heterologous protection: e.g., against drifted influenza virus strains.

## MATERIALS AND METHODS

### Viruses, recombinant proteins and antiserum generation.

The type A influenza virus (IAV) used in this study was the mouse-adapted H1N1 A/Belgium/1/2009 (Bel/09) strain (7). Bel/09 was propagated in Madin-Darby canine kidney (MDCK) cells in serum-free medium in the presence of TPCK (tosylsulfonyl phenylalanyl chloromethyl ketone)-treated trypsin (Sigma). The median tissue culture infective dose (TCID_50_) and median lethal dose (LD_50_) were calculated by the Reed and Muench method (31).

Recombinant soluble tetrameric Bel/09 NA (rNA) and trimeric Bel/09 HA (rHA) were produced as described previously (32). Briefly, the intracellular and transmembrane domains of NA as well as the stalk of NA were replaced by a type I secretion signal followed by a tetramerizing domain derived from *Tetrabrachion* (33). The HA trimer was stabilized with a trimerizing GCN4-derived leucine zipper (34). Secretion was facilitated by an N-terminal CD5-derived secretion signal, and a Strep-tag allowed purification from supernatants of transiently transfected HEK293T cells by affinity trap using a StrepTrap HP column followed by size exclusion chromatography in phosphate-buffered saline (PBS) using an AKTA explorer purification system (GE Healthcare Life Sciences).

One microgram of purified Bel/09 rNA or rHA was used per mouse per immunization step to produce mouse antiserum. Mice were primed and boosted 3 weeks apart in the presence of Sigma Adjuvant System (SAS), containing the immunostimulants monophosphoryl lipid A and synthetic trehalose dicorynmycolate. Mice were terminally bled via retro-orbital bleed, and serum was isolated by allowing clot formation.

### ELISA.

Wells of 96-well plates were coated overnight with rNA in either sodium bicarbonate or Dulbecco’s phosphate buffered saline (DPBS) plus 0.9 mM Ca^2+^ and 0.5 mM Mg^2+^ (Thermo Scientific) on Nunc MaxiSorp 96-well plates. The wells were blocked with 1% bovine serum albumin (BSA [Sigma]) in PBS. Monoclonal antibodies were applied, as indicated, in 0.5% BSA plus 0.1% Tween 20 in PBS (PBST). Horseradish peroxidase (HRP)-conjugated secondary antibodies, including goat anti-mouse total IgG (GE Healthcare), IgG2b, IgG1, IgG2a, IgG3, and IgE (Southern Biotech) or rabbit anti-human total IgG (Sigma) antibody was subsequently applied. 3,3′,5,5′-tetramethylbenzidine (TMB) substrate (BD) was added for detection, the reaction was stopped with 1 M H_2_SO_4_, and absorbance was read at 450 and 655 nm as a reference to correct for background.

### NI assays.

Antisera or monoclonal antibodies were tested for their ability to inhibit the activity of the viral NA using the standard enzyme-linked lectin assay (ELLA) essentially as described by Couzens et al. (35). Briefly, serial dilutions of heat-inactivated sera were incubated with Bel/09 at 70% maximal NA activity for 30 min at 37°C in PBS supplemented with 10 mg/ml BSA, 1 mM CaCl_2_, 0.5 mM MgCl_2_, and 0.5% Tween 20. Dilutions were applied to fetuin (50 μg/ml [Sigma-Aldrich])-coated wells of ELISA plates (Nunc) and incubated for 18 h at 37°C. HRP-coupled peanut agglutinin (PNA; 5 μg/ml [Sigma-Aldrich]) was used to detect galactose residues exposed after NA-mediated removal of sialic acid from fetuin.

### Mouse studies.

Six- to 8-week old female or male BALB/c mice (Charles River), *Fcer1g*^−/−^ BALB/c mice (Taconic), *Fcgr3*^−/−^ BALB/c mice, *Fcgr1*^−/−^ Fcgr3^−/−^ BALB/c mice and humanized FcγR mice (sourced from Jeffery Ravetch [24]) were housed under specific-pathogen-free conditions with food and water *ad libitum*. All mouse experiments complied with national (Belgian Law 14/08/1986 and 22/12/20333, Belgian Royal Decree 06/04/2010) and European legislation (EU Directives 2010/63/EU and 86/609EEG) on animal regulations. The ethics committee of the Vlaams Instituut voor Biotechnologie (VIB), Ghent University, Faculty of Science (Ethics Committee no. 2014-068 and 2016-059) approved all experiments.

Immune sera were passively transferred by intraperitoneal (i.p.) injection. Passive transfer of monoclonal antibodies N1-C4, N1-7D3, huN1-C4, and huN1-7D3 or huN1-C4 or huN1-7D3 F(ab′)_2_ fragments was performed by intranasal (i.n.) administration to sedated mice at equimolar amounts [1 or 2.5 mg/kg and 1.85 mg/kg for antibodies or F(ab′)_2_, respectively] in 50 μl. One day after serum or antibody transfer, the mice were infected i.n. with 0.1, 1, or 2 Bel/09 LD_50_s, as determined in WT mice, in a total volume of 50 μl under light isoflurane sedation. Mice were subsequently monitored for body weight change and survival and were euthanized if they lost ≥25% of their original body weight. In some experiments on days 3, 5, or 6, mice were sacrificed by cervical dislocation and lungs were excised. Lung homogenates were prepared and clarified as previously described (36), and viral titers were assessed by TCID_50_ assay.

### TCID_50_.

Standard TCID_50_ assays were used to assess viral titers in the clarified lung homogenates. The 96-well plates were seeded with MDCK cells, which were cultured in DMEM plus 10% fetal calf serum (FCS), and supplemented with nonessential amino acids, 2 mM l-glutamine, and 0.4 mM sodium pyruvate. Before infection, the cells were washed in serum-free medium and incubated with 10-fold dilutions of samples in serum-free DMEM containing 1 μg/ml of TPCK-treated trypsin (Sigma). Virus was detected in the wells by agglutination of chicken red blood cells after 7 days postinfection, and values were calculated by the Reed and Muench method (31).

### Generation of human chimeric monoclonal antibodies and F(ab′)_2_ fragments.

The variable heavy- and κ-light-chain regions of murine N1-C4 and 7D3 were synthesized as Homo sapiens codon-optimized gene fragments and cloned into a pTT5-based mammalian cell expression vector containing the human IgG1 heavy- and κ light-chain constant regions, respectively. Antibodies were produced by cotransfection 293T cells and purified from day 6 culture supernatants using a protein A affinity chromatography column.

Human N1-C4 and 7D3 were treated with IdeS enzyme (FragIT kit; Genovis) according to manufacturer’s instructions to generate F(ab′)_2_ fragments. Briefly, in 0.5-mg batches, antibodies were added to columns containing IdeS enzyme for 15 min. Flowthrough material was collected, and the CaptureSelect column (Fc affinity matrix) was used to negatively select F(ab′)_2_ fragments. Sodium dodecyl sulfate-polyacrylamide gel electrophoresis (SDS-PAGE) was used to monitor cleavage and purity of F(ab′)_2_ fragments, which were then used in mouse experiments.

### N-glycan analysis.

The N-glycan profile of the monoclonal antibodies was determined as previously described (37). N-glycans were prepared according to the “on-membrane” protocol. Briefly, 10 μg of monoclonal antibody was denatured and bound to a polyvinylidene fluoride (PVDF) membrane. The denatured proteins were reduced and carboxymethylated. After washing and blocking of the membrane, the N-glycans were released overnight at 37°C with 1.25 U (as defined by the International Union of Biochemistry and Molecular Biology) of PNGase F. The following day, the N-glycan samples were collected in a new Eppendorf tube and dried in a SpeedVac, followed by overnight labeling at 37°C with 5 μl labeling solution (a 1:1 [vol/vol] mixture of 20 mM APTS in 1.2 M citric acid and 750 mM picoline borane in dimethyl sulfoxide [DMSO]). The following day, the glycans were cleaned up over a Sephadex G10 resin and, after elution, analyzed by capillary electrophoresis on an ABI 3130 genetic analyzer. Fucosidase digests were performed on the cleaned-up glycans: 1 μl of the glycan mix was mixed with buffer and α-fucosidase (Glyko bovine kidney α-fucosidase; ProZyme) in a total concentration of 10 μl and incubated at 37°C overnight, followed by analysis by capillary electrophoresis.

### ADCC reporter bioassay.

The ADCC reporter bioassay core kit was purchased from Promega and used according to manufacturer’s instructions and as reported (38). Briefly, confluent monolayers of MDCK cells in a white tissue culture treated 96-well plate and infected with Bel/09 at an MOI of 1. Twenty-four hours later, infected cells were used as target cells in the ADCC reporter assay. Human monoclonal antibodies or the human IgG1 isotype control (BioXCell) were added in triplicate in a 3-fold dilution series starting at 100 μg/ml. ADCC bioassay effector cells (Jurkat V variant cells) were added, and plates were incubated at 37°C for 6 h. Bio-Glo luciferase assay reagent was added, and luminescence was measured. Fold induction was calculated from controls with buffer alone, and the 50% effective concentration (EC_50_) was determined in GraphPad Prism.

### Statistical analysis.

When comparing three or more sets of values, the data were analyzed by one-way ANOVA followed by *post hoc* analysis using Tukey’s multiple-comparison test. For changes over time, a two-way ANOVA was used. A log-rank test assessed survival significance. *P* values of <0.05 were considered significant. All analysis was performed using GraphPad Prism software.
